# SMARCA5 is required for the development of granule cell neuron precursors and Sonic Hedgehog Medulloblastoma growth

**DOI:** 10.1038/s41598-025-11857-3

**Published:** 2025-07-18

**Authors:** Foteini Tsiami, Layla Drwesh, Surender Surender, Julia Fitzgerald, Jens Schittenhelm, David J. Picketts, Rosalind A. Segal, Ghazaleh Tabatabai, Daniel J. Merk

**Affiliations:** 1 Department of Neurology and Interdisciplinary Neuro-Oncology, Hertie Institute for Clinical Brain Research, University Hospital Tübingen, Eberhard Karls University Tübingen, 72076 Tübingen, Germany; 2 Department of Neurodegeneration, University Hospital Tübingen, Hertie Institute for Clinical Brain Research, Eberhard Karls University Tübingen, 72076 Tübingen, Germany; 3 Institute of Neuropathology, Department of Pathology and Neuropathology, University Hospital Tübingen, Eberhard Karls University Tübingen, 72076 Tübingen, Germany; 4 Comprehensive Cancer Center Tübingen Stuttgart, University Hospital Tübingen, Eberhard Karls University Tübingen, 72076 Tübingen, Germany; 5 Regenerative Medicine Program, Ottawa Hospital Research Institute, Ottawa, ON K1H 8K6 Canada; 6 Department of Cancer Biology, Dana-Farber Cancer Institute, Boston, MA 02115 USA; 7 Department of Neurobiology, Harvard Medical School, Boston, MA 02115 USA; 8 Cluster of Excellence iFIT (EXC 2180) “Image Guided and Functionally Instructed Tumor Therapies”, Eberhard Karls University Tübingen, 72076 Tübingen, Germany

**Keywords:** Sonic Hedgehog Medulloblastoma, *SMARCA5*, Granule neuron precursors, Cerebellar development, Paediatric cancer, Development of the nervous system, CNS cancer

## Abstract

Medulloblastoma constitutes a molecularly diverse group of malignant embryonal brain tumors. Sonic hedgehog molecular group of medulloblastoma (SHH-MB) is a highly heterogeneous tumor entity, characterized by constitutive activation of the SHH signaling pathway. Due to lack of suitable cell line models, little is known about genetic dependencies in SHH-MB outside of the SHH pathway. By performing a CRISPR-Cas9 dropout screen in SMB21 cells derived from SHH-MB in *Ptch*^*+/−*^ mice, we aimed to identify genetic vulnerabilities in SHH-MB. Among the top scored gene hits, members of the SNF2-family of ATP-dependent chromatin remodelers including *Smarca5* emerged as genetic dependencies in SHH-MB, and we validate that *Smarca5* knockout inhibits SHH pathway activation and SHH-MB cell proliferation. Additional genetic ablation experiments *in vivo* revealed that conditional deletion of *Smarca5* in cerebellar granule cell neuron precursors (GCNPs), the cell origin of SHH-MB, significantly reduces the proliferative capacity of GCNPs and leads to cerebellar hypoplasia in mice. Furthermore, loss of *Smarca5* in GCNPs in an established mouse model of SHH-MB results in prolonged survival of tumor bearing mice. Our data underline the critical role of SMARCA5 during the development of the cerebellum and the pathogenesis of SHH-MB.

**Supplementary Information:**

The online version contains supplementary material available at 10.1038/s41598-025-11857-3.

## Introduction

Medulloblastoma (MB) is one of the most common malignant pediatric tumors of the central nervous system (CNS). Although standard-of-care regimens have increased overall survival rates of MB patients up to 70–80%^1^, they can induce long-term sequelae due to treatment toxicity. Thus, novel therapeutic strategies are needed. Profiling MB on a transcriptomic and proteomic level has revealed intertumoral heterogeneity, resulting in tumor stratification into four major consensus molecular subgroups: wingless (WNT), sonic hedgehog (SHH), Group 3 and Group 4^2,3^.

When it comes to SHH-driven MB, it accounts for approximately 30% of all MBs, while transcriptomics and DNA methylation analyses have defined four distinct SHH subtypes (SHH1-4) that differ in clinical as well as molecular features^4–6^. SHH-MB is suggested to derive from cerebellar granule neuron progenitor cells (GCNPs)^7^which depend on active SHH signaling^8,9^. During normal cerebellar development, GCNPs proliferate upon exposure to SHH in the external granule layer (EGL) and finally migrate inwards to form the internal granule layer (IGL) as mature granule neurons^10,11^. Somatic or germline alterations within the canonical SHH signaling pathway including *Smoothened* (*SMO*) and *Patched1* (*PTCH1*), promote the tumorigenic potential of GCNPs, resulting in aberrant pathway activation and eventually SHH-MB growth^7,12,13^.

Here, we made use of functional genomics as a powerful approach to interrogate gene function on a genome-wide scale in *in vitro* cancer models^14^. In a previous study, we employed a CRISPR-Cas9-based loss-of-function screen in a murine SHH-MB cell model (SMB21)^15^ in order to unravel genetic dependencies for this tumor entity, validating DNA methyltransferase 1 (DNMT1) as a druggable vulnerability in SHH-MB models^16^. Expanding the list of epigenetic modifiers that act as fitness genes in SHH-MB, we here reveal several Snf2-family proteins including *Smarca5* as genetic dependencies in SMB21 cells. We show that knockout of *Smarca5* in murine SHH-MB cells affects cell viability by blocking SHH signaling output. By genetically ablating *Smarca5* in GCNPs, we demonstrate that it is essential for proper cerebellar development and is required for formation of SHH-MBs in mice. Our data emphasize the essential role of chromatin remodelers in SHH-MB.

## Materials & methods

### Cell lines

SMB21 cells originate from SHH-MB tumors arising in *Ptch*^*+/−*^ mice, as previously described^15^. They grow as neurospheres in ultra-low attachment culture flasks (Corning) in DMEM/F12 medium (Corning) supplemented with 2% B27 and vitamin A (Thermo Fisher Scientific), 1% GlutaMax (Thermo Fisher Scientific) and 1% penicillin-streptomycin (Thermo Fisher Scientific). Prior to all experiments, cell seeding density was determined, in order to achieve optimal confluence, corresponding to 200,000 cells per ml. Cells were kept at 37^o^C humidity-controlled incubator with 5% CO_2_ and regularly tested for mycoplasma contamination.

### Ethics statement

All animal experimental procedures were approved by the regional council of Tuebingen and conducted according to animal welfare regulations (N10-21G license). All procedures were performed in accordance with the ARRIVE guidelines.

### Animals

*Math1-cre*^17^ and *SmoM2-YFP*^*Fl/Fl*^^18^ mice were obtained from Ulrich Schüller University Hospital Hamburg, Germany, while *Smarca5*^*Fl/Fl*^ mice^19^ were obtained Dr. David Picketts (Ottawa Hospital Research Institute, Canada). Genotyping was performed by PCR using ear genomic DNA. All mice were maintained on a 12-hour dark/light cycle and animals of both sexes were used for all experiments. In order to isolate whole brains, adult mice (≥ P21) were sacrificed by transcardiac perfusion, while pups at P5 by decapitation. Mice were anesthetized via intraperitoneal injection of narcotics that contained ketamine (120 mg/kg) and sedaxylan (10 mg/kg) diluted in 0.9% isotonic sodium chloride solution. Following anesthetics administration, inter-toe reflexes were checked and once negative, the abdominal cavity was opened, so the heart was visualized. A butterfly needle connected to ice cold Dulbecco´s phosphate-buffered saline was inserted into the left ventricle and the animals were perfused, until the liver turned white. Once perfusion was complete, brains were collected and processed for further histological analyses, as described below.

### CRISPR-Cas9 knockout dependency screen and analysis

Cas9-expressing SMB21 cells were screened with the Brie library (#73633, Addgene), which delivers 78,637 different sgRNAs targeting 19,674 murine genes^20^. Cells were transduced with predefined lentiviral volume by spinfection for 2 h at 930 g at 30^o^C and the next day, they were split into three technical replicates. A 500x library coverage was estimated to be achieved, meaning that on average each gRNA would be present in 500 cells. After selecting cells with 0.5 µg/ml puromycin for 5 days, they were propagated in culture for 21 days and finally at the last day of the screen genomic DNA was extracted from the surviving cells using the QIAamp DNA Blood Maxi Kit (QIAGEN). Genomic DNA was subjected to Next Generation Sequencing at the Broad Institute at MIT (Cambridge, USA).

For the analysis of the dropout screen, we first corrected log_2_ fold changes (LFC) of all sgRNAs in an unsupervised manner using *CRISPRcleanR*, in order to account for gene-independent responses to CRISPR-Cas9 editing^21^. To derive SMB21-context specific dependencies, we used the MAGeCK-RRA algorithm which identifies negatively selected sgRNAs in un unsupervised manner^22^as well as BAGEL2, which classifies essential and non-essential genes in a supervised manner^23^. Commonly depleted genes deriving from both methods at FDR < 5% are considered genetic vulnerabilities in SMB21 cells.

### Genetic validation *in vitro*

One sgRNA per *Smo* and *Smarca5* was generated and cloned into lentiCRISPRv2 puro vector (#98290, Addgene) according to the manufacturer´s protocol. sgRNA sequence for *sgSmo* is: 5´-CACCGGAACTCCAATCGCTACCCTG-3´ and for *sgSmarca5*: 5´-CACCGATGCTTCAAATGATTCGACA-3´. Lentivirus was produced in HEK293FT cells and SMB21 cells were transduced with lentiCRISPRv2-sgRNA plasmids by means of spinfection. In particular, 3 × 10^6^ cells were seeded in 2 ml per well in 12-well plates and approximately 500 µl virus was added on the wells. One well with no virus was used as non-infected control. Plates were centrifuged for 2 h at 930 g at 30^o^C and afterwards, 2 ml medium was added on top. Cells were incubated overnight. The next morning, cells were counted and seeded at their optimal density in flasks under 1 µl/ml puromycin. Selection ran for 3 to 5 days, until non-infected cells were all dead. Following puromycin selection, SMB21 sgRNA-transduced cells were seeded in triplicates at their optimal density in 100 µl/well in 96-well plates. Viability was determined at day 0, 3 and 7 by staining cells with CellTiter-Blue Reagent (Promega) and conducting cell measurements using plate reader GloMax (Promega). Viability at day 3 and 7 was normalized to day 0. Parental SMB21 cells were used as control cells.

### Genetic validation *in vivo*

In order to generate mice with a heterozygous and homozygous loss of *Smarca5*, we crossed *Smarca5*^*Fl/Fl*^ mice with *Math1-cre::Smarca5*^*Fl/+*^ mice^17,19^, resulting in mice of genetic background *Math1-cre:: Smarca5*^*Fl/+*^ and *Math1-cre:: Smarca5*^*Fl/Fl*^, respectively. Animals were sacrificed at P5 and P21 and *Math1-cre* mice were used as a control group. For the tumor study, *Math1-cre::Smarca5*^*Fl/+*^ mice were bred with *Smarca5*^*Fl/Fl*^::*SmoM2-YFP*^*Fl/Fl*^ mice^18,19^generating *Math1-cre::Smarca5*^*Fl/+*^::*SmoM2-YFP*^*Fl/+*^ and *Math1-cre::Smarca5*^*Fl/Fl*^::*SmoM2-YFP*^*Fl/+*^ mice. Animals were sacrificed either at P5 or monitored based on a stringent scoring sheet and sacrificed by transcardiac perfusion, when exhibiting neurological symptoms. *Math1-cre::SmoM2-YFP*^*Fl/+*^ mice were used as a tumor control group.

### Western blotting

Proteins were isolated from SMB21 parental and transduced cells using Pierce RIPA buffer (Thermo Fisher Scientific) with phosphatase inhibitors cocktail (1:100) (#5870, Cell Signaling). For the conventional western blotting, after mixing protein lysates with 4x LDS sample buffer for 10 min at 70^o^C, they were separated electrophoretically in 4–12% NuPage precast gels (Thermo Fisher Scientific) and transferred on nitrocellulose membranes. Then, the membranes were blocked with 5% nonfat dry milk-TBST buffer for 1 h at room temperature (RT) and incubated with primary antibodies overnight. The next day, the membranes were incubated with goat anti-rabbit (1:5000, ab97051, abcam) or goat anti-mouse (1:5000, ab97023, abcam) horse radish peroxidase (HRP)-conjugated secondary antibodies. Cheluminescence detection of protein bands was performed in ChemiDoc imaging machine (BioRad) using solution kit SuperSignal West Dura (Thermo Fisher Scientific). The following primary antibodies were used at indicated dilutions: GLI1 (1:1000, #2534, Cell Signaling), SMARCA5 (SNF2H, 1:2000, ab72499, abcam), GAPDH (1:1000, #2118, Cell Signaling) and β-tubulin (1:1000, #86298, Cell Signaling). GLI1 and SMARCA5 protein levels were quantified using ImageJ v2.9.0 (https://imagej.net/software/fiji/downloads) and normalized to corresponding β-tubulin or GAPDH levels of each sample.

For the automated western blotting, whole cell lysate (WCL) samples were analyzed using the JESS Simple Western™ system (ProteinSimple^®^, Bio-Techne) with the 12–230 kDa separation module and an 8 × 25 capillary cartridge (#SM-FL004). DTT, biotinylated molecular weight ladder, and fluorescent standards (EZ standard pack I #PS-ST01EZ-8) were reconstituted in double-distilled water (ddH_2_O) according to the manufacturer’s instructions. Lysates were mixed with 1x fluorescent master mix and diluted to a final protein concentration of 0.4–0.8 mg/mL using 0.1x sample buffer (from 10x stock solution, #042–195). Samples were denatured at 95 °C for 5 min before loading. A total of 4 µL (containing 1.6–3.2 µg protein) was loaded per well. The following antibodies with the indicated dilutions were used for analyzing the lysates: Smo (#sc-166685, Santa Cruz) at 1:10, Smarca5 (SNF2H, 1:2000, ab72499, abcam) and GAPDH (1:1000, #2118, Cell Signaling) at 1:30. Samples were decorated with the antibodies in one probe and total protein stain (#DM-TP01) in the second probe using sequential detection by RePlex™ reagent kit (#RP-001). Samples were decorated with ready to use goat anti-rabbit secondary HRP-conjugated antibody (#042–206) and with ready to use goat anti-mouse secondary HRP-conjugated antibody (#042–205). For preparing the final primary antibody dilution mix, milk-free antibody diluent buffer 2 was used (#042–203). Protein signal was developed using enhanced chemiluminescence (ECL) reagents provided in the secondary antibody module (#DM-001) and according to the manufacturer´s instructions. Data acquisition and analysis were performed using Compass for Simple Western (SW) software v7.0 (https://www.bio-techne.com/resources/instrument-software-download-center/compass-software-simple-western#version7). Signal quantification was based on the area under the curve (AUC) of electropherogram peaks from the high dynamic range 4.0 generated for each target protein. For normalization, total protein staining was used as a loading control. The system’s in-capillary total protein detection module labeled all proteins in the separation matrix, allowing lane-by-lane normalization. Target protein signals were normalized to the total protein signal from the same capillary to account for variations in sample loading. Normalized quantification data were exported from Compass software to GraphPad Prism 9 for statistical analysis and figure generation.

### Flow cytometry

SMB21 cells were dissociated into a single-cell suspension using accutase, followed by centrifugation at 700 × g for 5 min. The resulting pellet was then washed with PBS, and after discarding the supernatant, cells were resuspended in a primary antibody cocktail (10 µL SMO (#sc-166685, Santa Cruz) in 100 µL FACS buffer (PBS supplemented with 2% FCS and 0.5 M EDTA). Cells were incubated at 4 °C for 1 h in the dark. Afterwards, the cells were washed and subsequently incubated with secondary antibody (Goat anti-Mouse IgG (H + L) Cross-Adsorbed Secondary Antibody, Alexa Fluor™ 488; invitrogen #A-11001) for 45 min at 4 °C, in the dark, followed by two additional washes with FACS buffer (5 min at 700 × g). Finally, cells were resuspended in 200 µL FACS buffer and analyzed on a MACSQuant Analyzer (Miltenyi Biotec). Data analysis was performed using FlowJo software (version 10.0). A minimum of 100,000 events were recorded per sample and Fluorescence minus one (FMO) controls were employed for accurate gating.

### Histology and immunohistochemistry

Dissected mouse brains were cut in the midline, snap-frozen in liquid nitrogen, fixed in Tissue-Tek medium (O.C.T, Sakura Finetek) and cut sagittally at 8 μm thickness using the cryotome (LEICA CM 3050 S). For Hematoxylin and eosin (H&E) staining, slides were first fixed in acetone (-20^o^C) and 80% methanol (4^o^C), followed by a 5-minute staining with 0.1% hematoxylin (SIGMA-ALDRICH). After counterstaining with 1% eosin (Care Roth) for 2 min, slides were passed through a graded series of ethanol. For the luxol fast blue (LFB) staining, sections were fixed in 4.5% formalin for 5 min, incubated with LFB staining solution overnight (~ 55^o^C) and counterstained with 0.1% cresyl violet.

For the immunohistochemistry protocol, sections were fixed either on 4% PFA (RT) or acetone (-20^o^C) and 80% methanol (4^o^C), depending on the antibody used. First, slides were blocked in 10% BSA in PBS-Tween 0.3% for 1 h and then incubated with primary antibody diluted in 2% BSA in PBS-Tween 0.06% overnight at 4^o^C. The following day, slides were incubated with horse anti-rabbit (H + L, BA-1100, Vector Laboratories) or goat anti-mouse (H + L, BA-9200, Vector Laboratories) IgG secondary biotinylated antibodies diluted at 1:400 in 2% BSA in PBS-Tween 0.06% for 1 h at RT. After incubating them with avidin/biotin-based peroxidase solution (VECTASTAIN Elite ABC) for 30 min, slides were stained with NovaRed Substrate-HRP solution (Vector Laboratories) for up to 5 min and counterstained with hematoxylin for 45 s. Finally, the slides were dehydrated with graded ethanol. The following primary antibodies were used: Smarca5 (Snf2h) (1:200, ab72499, abcam), Cre (1:100, #15036, Cell Signaling), Ki67 (1:100, ab16667, abcam), Cleaved Caspase 3 (1:100, #9664, Cell Signaling), Pax6 (1:400, ab19045, abcam) and NeuN (1:400, #24307, Cell Signaling). Fraction of antibody-positive cells to the total number of cells in the region of interest was calculated using ImageJ software. All images were acquired using bright-field microscopy (Zeiss, Axioplan 2) in the Department of Cellular Neurology (Tübingen, Germany) and analyzed using Adobe Photoshop CS5.1 or Gimp (2.10.36).

### Statistical analysis

All statistical analyses were performed in GraphPad Prism 9 or R studio (4.0.5). One-way ANOVA with Tukey´s multiple comparisons test was conducted for comparing two or more groups, while Fisher´s exact test was used for the statistical analysis of cell quantifications. For the analysis of Kaplan-Meier curves, log-rank (Mantel-Cox) test was performed.

## Results

### Dropout screen unravels *Smarca5* as an SMB21-context specific vulnerability

**Fig. 1:**
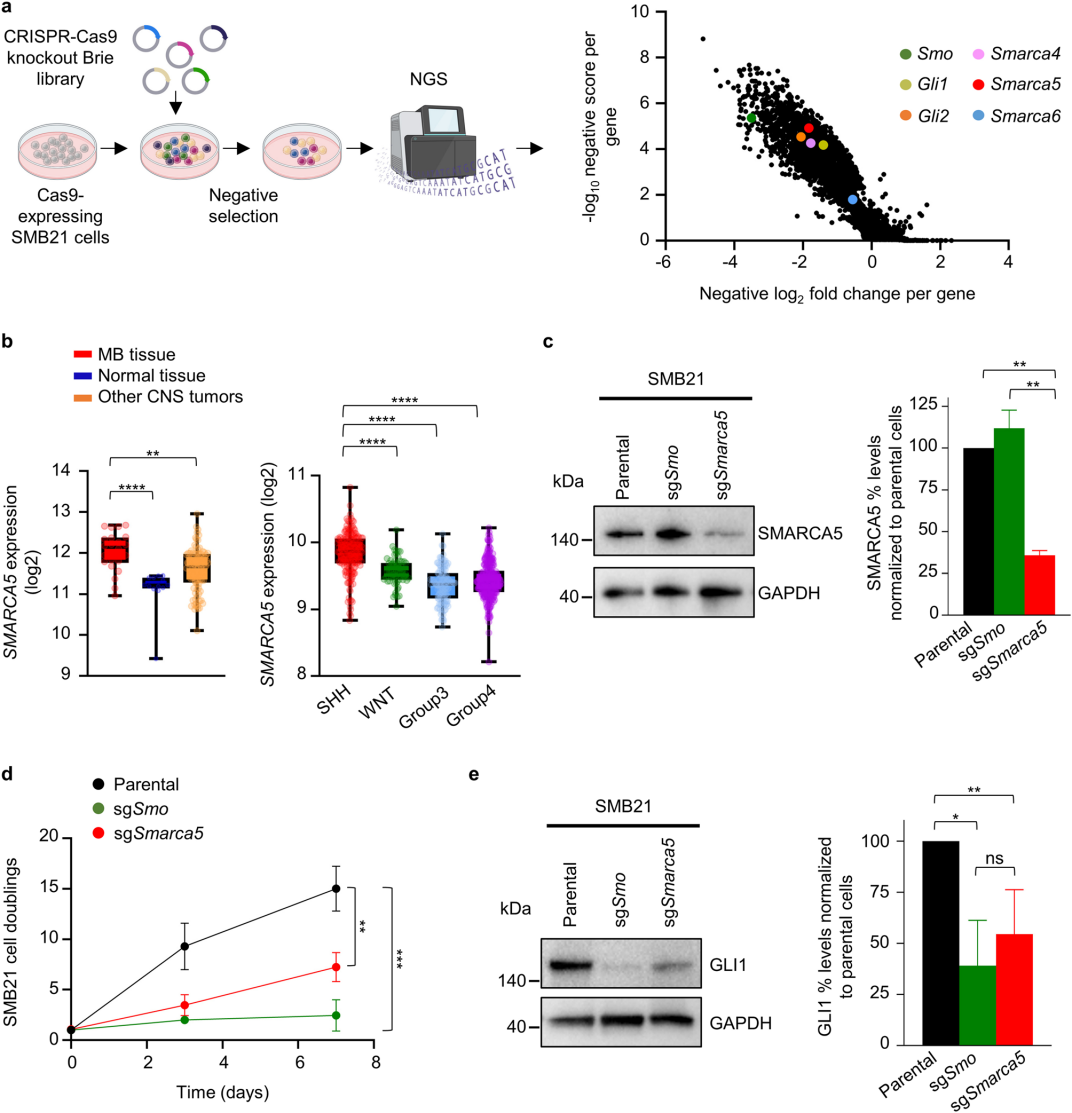
CRISPR-Cas9 knockout screen identifies *Smarca5* as genetic dependency for SMB21 cells. (**a**) Illustration of CRISPR-Cas9 negative selection screening timeline for SMB21 cells. Illustration created with Biorender.com. Volcano plot showing the log_2_ fold change per gene and their associated negative score as determined by MAGeCK-RRA. Selected dependencies are denoted as colored data points. (**b**) Box plots demonstrating *SMARCA5* expression in MB, CNS tumors and normal tissue, as determined from Griesinger (left panel) and Cavalli (right panel) publicly available datasets. Whiskers represent minimum and maximum points across all data points per group. The confidence intervals of the means for both plots are as follows: CI_MB_= 11.82–12.24, CI_Normal tissue_= 10.82–11.45, CI_Other tumors_= 11.48–11.69, CI_SHH_= 9.787–9.882, CI_WNT_= 9.516–9.620, CI_Group3_= 9.307–9.407 and CI_Group4_= 9.396–9.460. One-way ANOVA, Tukey´s multiple comparisons test. (**c**) Western blotting depicting SMARCA5 protein expression in sg*Smo*, sg*Smarca5*, and control SMB21 cells (left panel). Corresponding bar graph illustrates quantification of SMARCA5 (right panel). (**d**) Analysis of cell population doublings of SMB21 cells transduced with the indicated sgRNAs, as compared to SMB21 parental cells over 7 days. Two-way ANOVA, Tukey´s multiple comparisons test (*n* = 4). (**e**) Western blotting depicting GLI1 protein levels in sg*Smo*, sg*Smarca5*, and control SMB21 cells (left panel). Quantification of GLI1 protein levels in cells shown is indicated in the right panel. One-way ANOVA, Tukey´s multiple comparison test (*n* = 4). All graphs display mean ± SD. *p ≤ 0.05, ** *p* ≤ 0.01, **** *p* ≤ 0.0001.

We conducted a genome-wide CRISPR-Cas9 knockout screen in a previously described murine SHH-MB cell model, SMB21^15,16^, using the Brie library which targets 19,674 coding genes^20^ (Fig. 1a). We assessed gene essentiality by combining supervised (BAGEL2)^23^ and unsupervised (MAGeCK-RRA)^22^ tools. Known core constitutive essential genes (CCEs) exhibited high negative scores, while non-essentials mostly remained unaffected in our screen (Supplementary Fig. S1a). After subtracting CCEs genes^24^we defined 2,211 SMB21-context specific dependencies at FDR < 5% (Supplementary Table S1). Representation on the sgRNA level revealed robust depletion of sgRNAs targeting critical members of the SHH pathway including *Smo* and *Gli2*, providing further evidence that SMB21 cells depend on SHH pathway for their survival, as we previously described^16^ (Supplementary Fig. S1b).

In a previous study, we validated the epigenetic modifier DNA methyltransferase 1 (DNMT1) as a druggable dependency in SMB21 cells^16^and here aimed to expand our knowledge of epigenetic regulators and their role during SHH-MB development and progression. Of note, we observed that several members of the epigenetic machinery scored strongly in the screen (Fig. 1a, Supplementary Fig. S1b), including Snf2-protein family members of chromatin remodelers *Smarca4*, *Smarca5* and *Smarca6*. Previous studies have shown that *Smarca5* and *Smarca4* are essential for GCNP proliferation^19,25^while *SMARCA6* displays high expression levels in the murine cerebellum and SHH-MB, acting as a downstream effector of the SHH pathway^26^. Additionally, we interrogated publicly available microarray data in regard to *SMARCA5* expression in human MB tissue. According to these data^27^*SMARCA5* expression is significantly higher in MB patients than normal tissue and other CNS tumors (Fig. 1b), while across different molecular MB subgroups^6^SHH subgroup displayed highest gene expression, suggesting a role for *SMARCA5* in SHH-MB. Although no correlation was observed between survival outcome and high or low *SMARCA5* expression in any of the four MB subgroups, within different SHH-subtypes lowest *SMARCA5* expression was observed in SHH-γ patients, which are associated with a better survival outcome when compared to β subtype^6^ (Supplementary Fig. S2a, b). Based on these preliminary data, we sought to address the role of *Smarca5* both during normal cerebellar development and the progression of SHH-MB.

To further validate the screen findings, we used a CRIPSR-Cas9-based approach to generate *Smarca5* knockout SMB21 cell (*sgSmarca5*), and used *Smo* knockout cells (*sgSmo*) as an internal positive control. First, we evaluated the *Smarca5* knockout efficacy via conventional and automated western blotting analysis, revealing a more than 50% reduction in SMARCA5 protein expression in *Smarca5* knockout SMB21 cells (Fig. 1c, Supplementary Fig. S3a-c). Capillary-based western blotting using a SMO antibody detected proteins at three different sizes, all of which were strongly depleted in *Smo* knockout cells (Supplementary Fig. S3b-c), and this was further verified by flow cytometry analysis (Supplementary Fig. S3d). Next, we assessed SMB21 viability upon *Smarca5* knockout. As compared to SMB21 parental cells, *Smarca5* knockout significantly reduced cell proliferation, similar to loss of *Smo* (Fig. 1d), supporting our screen findings that *Smarca5* is required for SHH-MB tumor cell growth. Moreover, we show that *Smarca5* knockout significantly suppressed protein expression of the positive SHH regulator GLI1, similar to *Smo* knockout, providing evidence that *Smarca5* affects SHH signaling output (Fig. 1e, Supplementary Fig. S3e). Interestingly, *Smarca5* knockout cells also displayed a significant reduction in SMO protein expression as deduced from the flow cytometry analysis (Supplementary Fig. S3d), further suggesting that *Smarca5* impacts SHH pathway.

### Loss of *Smarca5* disrupts murine cerebellar development and mitigates tumor proliferation in SHH-MB mice

**Fig. 2:**
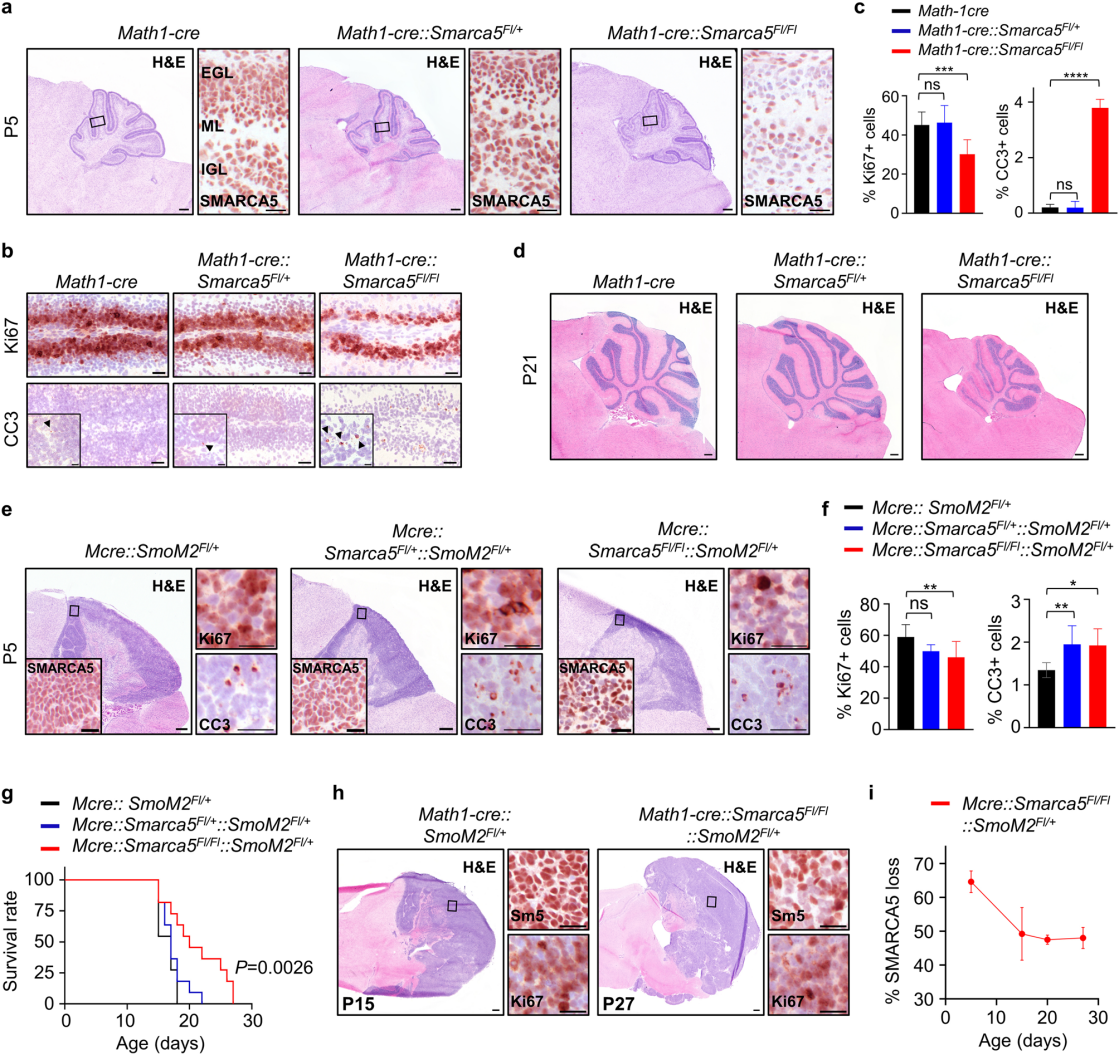
Loss of *Smarca5 in vivo* during normal cerebellar development and SHH-MB formation. (**a**) H&E staining and immunohistochemistry for SMARCA5 in the cerebellum of *Math1-cre* (left panel), *Math1-cre::Smarca5*^*Fl/+*^ (middle) and *Math1-cre::Smarca5*^*Fl/Fl*^ (right panel) mice at P5. (**b**) Immunohistochemistry for Ki67 and Cleaved caspase 3 (CC3) in the EGL of cerebella from mice with indicated genotypes at P5. Black arrowheads indicate cleaved Caspase-3 positive cells. (**c**) Quantification of Ki67 and cleaved Caspase 3-positive cells in the EGL of mice shown in (b) (*n* = 4, Fisher´s exact test). (**d**) Representative H&E stainings of whole cerebella from *Math1-cre* (left panel), *Math1-cre::Smarca5*^*Fl/+*^ (middle panel) and *Math1-cre::Smarca5*^*Fl/Fl*^ (right panel) mice at P21. (**e**) Representative H&E stainings of cerebellar tumors and immunohistochemistry for SMARCA5, Ki67 and cleaved Caspase 3 in tumors from mice with indicated genotypes at P5. (**f**) Quantification of Ki67 (left) and cleaved Caspase 3 (right) positive cells in tumors from mice shown in (e) (*n* = 3, Fisher´s exact test). (**g**) Kaplan-Meier curves showing survival of *Math1-cre::SmoM2*^*Fl/+*^ (*n* = 11), *Math1-cre::Smarca5*^*Fl/+*^::*SmoM2*^*Fl/+*^ (*n* = 11) and *Math1-cre::Smarca5*^*Fl/Fl*^::*SmoM2*^*Fl/+*^ (*n* = 11) mice. Significance in survival is indicated using log rank (Mantel-Cox) test. (**h**) H&E-stained cerebellar tumors from *Math1-cre::SmoM2*^*Fl/+*^ (left) and *Math1-cre::Smarca5*^*Fl/Fl*^::*SmoM2*^*Fl/+*^ (right) mice at different endpoints. Immunohistochemistry for SMARCA5 (Sm5) (upper panel) and Ki67 (lower panel) in tumors from these mice are indicated. (**i**) Fraction of SMARCA5-negative cells in cerebellar tumors from *Math1-cre::Smarca5*^*Fl/Fl*^::*SmoM2*^*Fl/+*^ mice from P5 to P27. 4x magnification, scale bar, 500 μm; 20x magnification, scale bar, 50 μm; 40x magnification, scale bar, 20 μm. EGL, external granular layer; ML, molecular layer; IGL, internal granular layer. All graphs display mean ± SD. *p ≤ 0.05, ** *p* ≤ 0.01, *** *p* ≤ 0.001, **** *p* ≤ 0.0001.

Having shown that *Smarca5* is essential for SHH-MB proliferation in vitro, we next aimed to evaluate its role in the development of normal murine cerebellum. It has been previously demonstrated that *Smarca5* knockout murine embryos die during the peri-implantation stage, indicating that *Smarca5* is required for proliferation of early blastocyst-derived stem cells and adult human hematopoietic progenitors, highlighting its crucial role at a very early stage in development^28^. Furthermore, conditional *Smarca5* knockout in cerebellar progenitors has been shown to induce GCNP cell death, leading to cerebellar hypoplasia and severe motor deficits in adult mice^19^. Using the same conditional *Smarca5* allele, we here used a Math1-directed Cre driver line to ablate *Smarca5* expression in GCNPs during early murine development^19,29^. In this mouse model, Cre recombination results in excision of exon 5 of *Smarca5* which encodes the evolutionary conserved ATP-binding pocket critical for remodeling activity. We confirmed that SMARCA5 is highly expressed in proliferating GCNPs of the EGL at P5, as well as mature granule neurons of the IGL in the murine cerebellum of *Math1-cre* and *Math1-cre::Smarca5*^*Fl/+*^ mice at P5 and P21 (Fig. 2a, Supplementary Fig S4a, Supplementary Fig. 5Sb). These findings support previous studies reporting predominant SMARCA5 expression in proliferating neurons during early postnatal development^30 with ^peak expression in proliferating GCNPs at P7, and attenuated but continued expression in adult cerebella^19^. We show that SMARCA5 expression was specifically lost in proliferating GCNPs in the EGL in the anterior part of the cerebellum of *Math1-cre::Smarca5*^*Fl/Fl*^ mice at P5, while its expression was maintained in the posterior region (Fig. 2a and Supplementary Fig. S4a). This observation is likely due to the well-known anterior-to-posterior generation of GCNPs in the rhombic lip during embryonal development^29^suggesting that recombination before embryonic day E15 is much more efficient than at later time points. Furthermore, we find Cre expression predominantly in the EGL and few cells of the IGL in *Math-cre* mice at P5, and Cre-positive cells in the EGL of *Math-cre::Smarca5*^*Fl/Fl*^ mice are virtually all SMARCA5-negative in anterior lobes, suggesting successful recombination in Math1-positive GCNPs (Supplementary Fig. S4b). Phenotypically, we show that *Math1-cre::Smarca5*^*Fl/Fl*^ mice exhibited a significant reduction in proliferation and a concomitant increase in apoptotic activity in GCNPs when compared to control mice, while no difference was observed in mice with a heterozygous loss of *Smarca5* (Fig. 2b, c). In line with these results, H&E and LFB stainings of whole cerebella revealed severe hypoplasia in *Math1-cre::Smarca5*^*Fl/Fl*^ mice at P21 (Fig. 2d, Supplementary Fig. S5a, b). A significant increase of NeuN- and Pax6-positive cells was detected in the molecular layer of *Math1-cre::Smarca5*^*Fl/Fl*^ mice at P21 (Supplementary Fig. S5b, c), suggesting that a subset of differentiated granule neurons accumulated in the molecular layer. While not tested here in detail, this might be due to a defect in migration of *SMARCA5*-deleted GCNPs. Actively proliferating cells were not observed in any of the investigated mouse models at P21 (Supplementary Fig. S5b). In summary, we show that loss of *Smarca5* interferes with GCNP proliferation, resulting in cerebellar hypoplasia in mice.

Next, we asked whether loss of *Smarca5* affects SHH-MB formation *in vivo*. Using an established mouse model of SHH-MB that drives constitutive expression of a mutated form of *Smo* in GCNPs^18^we generated SHH-MB mouse models that harbor a heterozygous (*Math1-cre::Smarca5*^*Fl/+*^::*SmoM2*^*Fl/+*^) or homozygous deletion (*Math1-cre::Smarca5*^*Fl/Fl*^::*SmoM2*^*Fl/+*^) of *Smarca5* (Fig. 2e). Via immunohistochemistry we observed reduced SMARCA5 expression in cerebellar tumors of *Math1-cre::Smarca5*^*Fl/Fl*^::*SmoM2*^*Fl/+*^ mice (Fig. 2e, Supplementary Fig. S6a), which displayed a significant decrease in proliferating tumor cells in the cerebellum at P5 as compared to control tumor mice (Fig. 2f). Interestingly, apoptotic activity in tumor areas was increased both in mice with a heterozygous as well as a homozygous loss of *Smarca5* as compared to control tumor mice. However, tumor mice with a heterozygous deletion of *Smarca5* did not show a significantly prolonged survival as compared to control tumor mice, while mice with a homozygous *Smarca5* deletion displayed a significant survival benefit (Fig. 2g). Histological examination of cerebellar tumors showed that *Math1-cre::Smarca5*^*Fl/Fl*^::*SmoM2*^*Fl/+*^ mice did not display any apparent difference in tumor morphology when compared to tumor control mice despite their difference in survival outcome (Fig. 2h, Supplementary Fig. S6b). In that regard, we determined the efficacy of *Smarca5* loss by quantifying the fraction of SMARCA5-negative cells to total tumor cells via immunostaining. While we had noticed insufficient recombination of the *Smarca5* locus in a subset of tumor cells at early stages (Fig. 2e), we found that the fraction of SMARCA5-negative cells decreased during postnatal tumor development (Fig. 2i). These data suggest that SMARCA5-negative cells are negatively selected during tumor development, further supporting the essential role of this chromatin modifier for growth of SHH-MB.

## Discussion

Medulloblastoma comprises a heterogeneous group of embryonal tumors of the central nervous system, stratified into four distinct main molecular groups, which display differential transcriptional, proteomic, as well as epigenetic features. Within SHH-MB, further molecular subgroups have been delineated, emphasizing the intertumoral complexity characterizing these tumors. Gaining insight into the genetic events that lead to tumorigenesis will lay the basis for more efficacious targeted therapies for this tumor entity.

By performing a genome-scale CRISPR-Cas9 knockout screen, we deciphered genetic vulnerabilities in a previously described murine SHH-MB cell model, SMB21^15^. Our downstream bioinformatic analysis revealed a robust depletion of members of ATP-dependent chromatin remodelers including SMARCA5, a member of the ISWI subfamily. Given the lack of stable, human cell models faithfully representing SHH-MB, our downstream genetic validations were performed exclusively in murine cells, SMB21. Through genetic validation experiments, we show that SMARCA5 is required for SMB21 proliferation by affecting SHH signaling output. Moreover, interrogation of publicly available datasets demonstrated that *SMARCA5* expression in SHH-MB is higher than in all other MB molecular groups. SMARCA5 is known to be crucial for mammalian embryonal development, as germline knockout results in embryonic lethality in mice^28^. Through genetic validation, we show that while loss of *Smarca5* alone in GCNPs, the proposed cell origin of SHH-MB^7^does not induce tumor formation in mice, it rather affects their proliferative capacity during early postnatal development and results in increased apoptotic activity, thus leading to a severe cerebellar phenotype in mice. Our findings are well in line with a previous study reporting GCNP cell death and cerebellar hypoplasia upon conditional knockout of *Smarca5* in mice^19^altogether validating the role of SMARCA5 in the developing cerebellum.

Moreover, while previous studies have addressed the role of *Smarca5* during murine development, here for the first time we investigated its function in SHH-MB growth. Our data suggest that loss of *Smarca5* significantly extends the survival time of SHH-MB mice, proposing a critical role for *Smarca5* in formation of SHH-MBs, being in line with high *SMARCA5* expression in SHH-MB patients. Of note, from P5 to P27 we observed a reduction in SMARCA5-negative cells in *Math1-cre::Smarca5*^*Fl/Fl*^::*SmoM2*^*Fl/+*^ mice, further supporting the essential function of *Smarca5* in SHH-MB. This finding indicates that differences in survival between *Math1-cre::Smarca5*^*Fl/Fl*^::*SmoM2*^*Fl/+*^ and *Math1-cre::SmoM2*^*Fl/+*^ mice would have been more prominent with a higher penetrance of *Smarca5* knockout in our mouse model. Besides *Smarca5*, other SNF2-family members of ATP-dependent chromatin remodelers such as *Smarca4* and *Smarca6* also scored highly in our screen, further corroborating previous studies showing their involvement in cerebellar and SHH-MB development^25,26,31^. Large scale genomic studies have revealed alterations of the epigenome across all MB subgroups, which often appear to be subgroup-specific^32–36^. Particularly, transcriptional profiling of SHH-MB has demonstrated that chromatin organization is affected in SHH-MB patients, with oncogenic alterations in chromatin modulation pathways occurring more frequently in adult patients^35,36^. *CREBBP*, a transcriptional co-activator, has also been described to drive SHH-MB growth *in vivo*^37^further indicating that the epigenetic machinery is involved in SHH-MB development.

Several *omics* studies have shed light on the significant role that chromatin modulators play in the pathogenesis of medulloblastoma that could potentially open up new opportunities for alternative therapeutic regimens. Using functional genomics in a faithful *in vitro* model of SHH-MB and mouse models, we provide evidence that SMARCA5 is essential for the regulation of SHH activation, GCNP survival, and SHH-driven MB growth. Thus, our data shed new light on the role of chromatin remodelers in the development of SHH-MB.

## Electronic supplementary material

Below is the link to the electronic supplementary material.

Supplementary Material 1

Supplementary Material 2

## Data Availability

Original raw read counts and quality metrics from PoolQ for SMB21 CRISPR-Cas9 knockout screen can be found at figshare (https://doi.org/10.6084/m9.figshare.25992037.v1). All major code from this study regarding CRISPR screening data analysis are available on zenodo as a GitHub release (https://zenodo.org/doi/10.5281/zenodo.11547189). For further information, contact the corresponding author.
