# Supplementary material for: SMARCA5 is required for the development of granule cell neuron precursors and Sonic Hedgehog Medulloblastoma growth
Source: Sci Rep. 2025 Jul 18;15:26091. doi: 10.1038/s41598-025-11857-3 (PMC12274346; doi:10.1038/s41598-025-11857-3)
Supplement: Supplementary file 1 — Supplementary Material 1 [file 41598_2025_11857_MOESM1_ESM.pdf]

Title

SMARCA5 is required for the development of granule cell neuron precursors and sonic hedgehog medulloblastoma growth

Authors

Foteini Tsiami, Layla Drwesh, Surender Surender, Julia Fitzgerald, Jens Schittenhelm, David J. Picketts, Rosalind A. Segal, Ghazaleh Tabatabai, and Daniel J. Merk*

*corresponding author

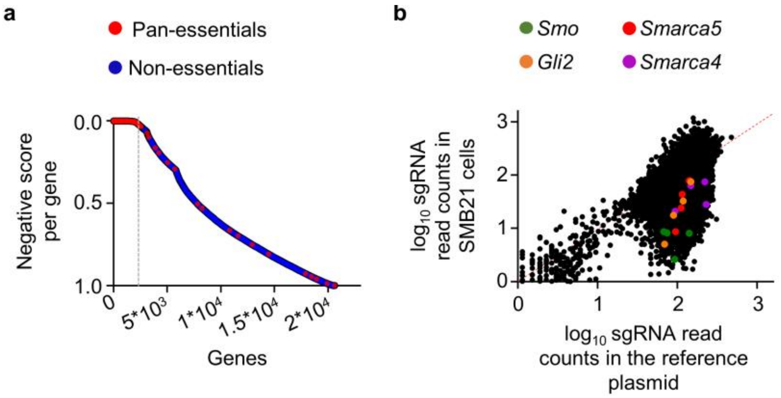

Figure S1. CRISPR-Cas9 knockout screen analysis in SMB21 cells. (**a**) Ranking plot depicting negative scores per gene as determined by MAGeCK-RRA. Red data points represent pan-essential genes and blue ones non-essentials. Dashed line indicates a 5% FDR cut. (**b**) Scatter plot of sgRNA representation in the screen compared to the reference plasmid. Colored data points correspond to different gRNAs targeting hit genes.

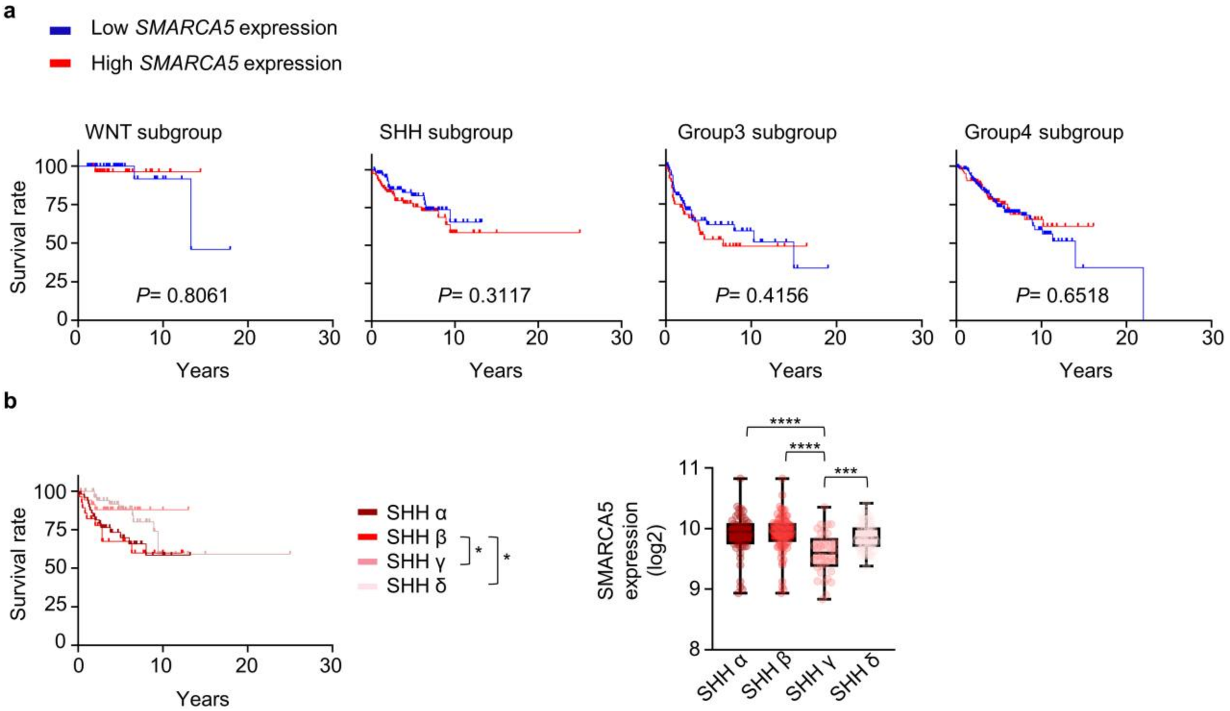

Figure S2. Interrogation of Cavalli survival and microarray data in regard to *SMARCA5*. (**a**) Kaplan-Meier curves of SHH-, WNT-, Group3- and Group4-MB patients with low (blue data points) and high (red data points) *SMARCA5* expression. (**b**) Survival analysis of MB patients assigned to four SHH-subgroups (left) and box plot illustrating their corresponding *SMARCA5* expression (right). Whiskers in the box plot represent minimum and maximum points across all data points per group (One-way ANOVA, Tukey´s multiple comparisons test). The confidence intervals of the mean per group are as follows: $CI_{SHH\text{-}\alpha}$= 9.797-9.996, $CI_{SHH\text{-}\beta}$= 9.850-9.991, $CI_{SHH\text{-}\gamma}$= 9.472-9.710 and $CI_{SHH\text{-}\delta}$= 9.809-9.921. Log rank (Mantel-Cox) test has been performed for the statistical analysis of all survival curves and corresponding *P* values are denoted. All graphs display mean ± SD. *p ≤ 0.05, *** p ≤ 0.001, **** p ≤ 0.0001.

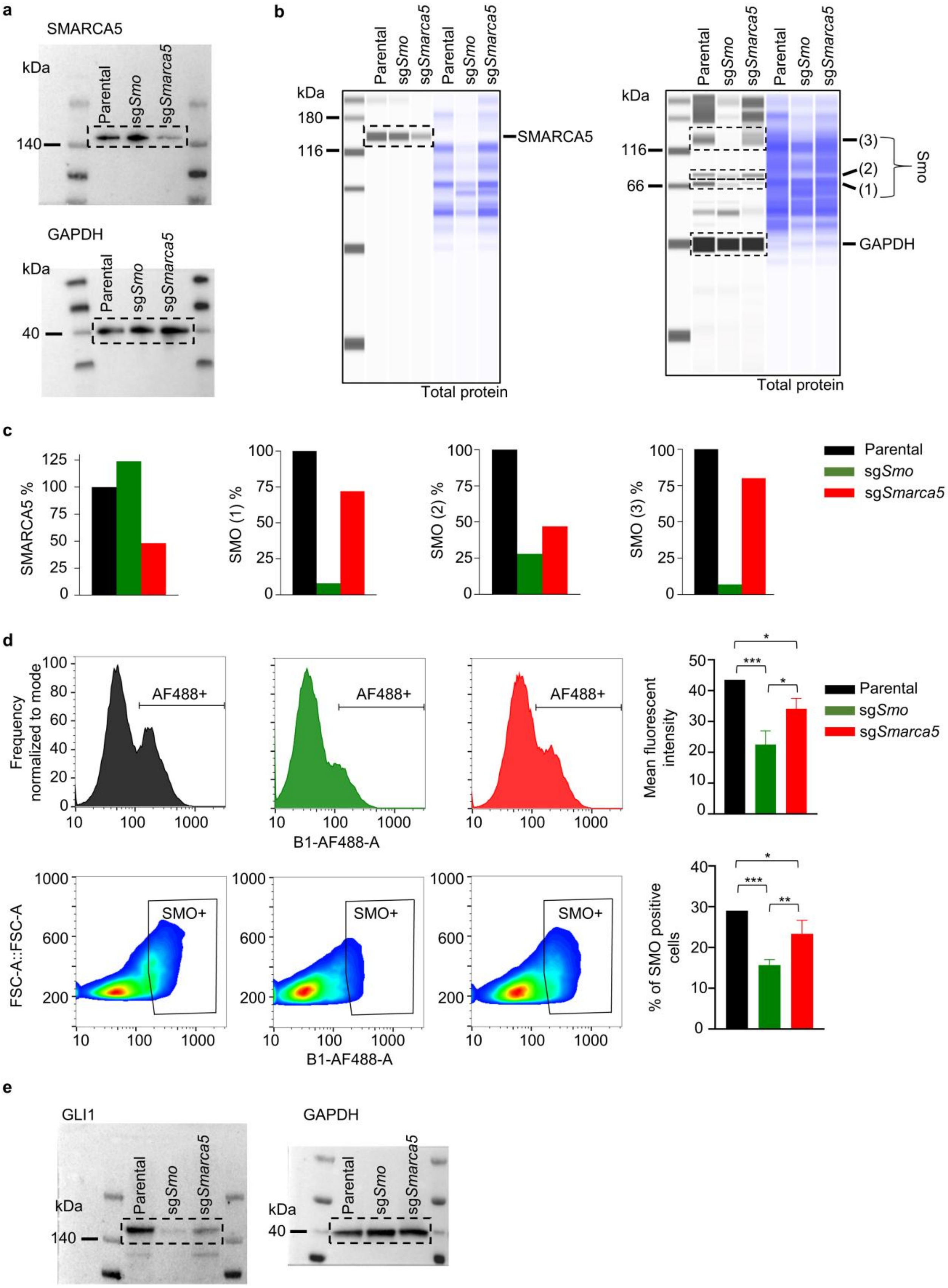
a
SMARCA5
kDa
Parental
sgSmo
sgSmarca5
140
GAPDH
kDa
Parental
sgSmo
sgSmarca5
40
b
Parental
sgSmo
sgSmarca5
Parental
sgSmo
sgSmarca5
kDa
180
116
SMARCA5
Total protein
kDa
116
66
(3)
(2)
(1)
Smo
GAPDH
Total protein
c
SMARCA5 %
SMO (1) %
SMO (2) %
SMO (3) %
Parental
sgSmo
sgSmarca5
d
Frequency normalized to mode
AF488+
B1-AF488-A
Mean fluorescent intensity
FSC-A::FSC-A
SMO+
% of SMO positive cells
e
GLI1
kDa
140
Parental
sgSmo
sgSmarca5
GAPDH
kDa
40

Figure S3. Protein expression of SMARCA5, SMO, and GLI1 in sg*Smo*, sg*Smarca5*, and control SMB21 cells. (**a**) Whole membrane images of SMB21 cells blotted for SMARCA5 and GAPDH antibodies. Samples of interest as illustrated in Fig. 1c are indicated by the black dotted frame. (**b**) Whole lane-by-lane in-capillary SMARCA5 (left panel) and SMO (right panel) detection in the indicated cell lines. Total protein detection per antibody is also depicted. SMO (1), SMO (2) and SMO (3) bands correspond to approximately 69, 82 and 136 kDa. (**c**) Bar graphs illustrating relative levels of SMARCA5, SMO (1), SMO (2) and SMO (3) proteins shown in (b) in sg*Smo* and sg*Smarca5* cells as compared to control cells. Normalization was done based on total protein levels per sample. (**d**) Upper panel: Histograms illustrating Alexa Fluor488-positive signal and frequency of positive events for SMO, normalized to mode per cell line. Bar graph depicts mean fluorescent intensity signal against SMO antibody. Lower panel: Density plots representing the distribution of cells form negative to positive detection against SMO antibody gated for SMO positive events. Black frame highlights SMO positive cell events. Bar graph illustrates percentage of SMO positive cells, as shown in the density plots (One-way ANOVA, Tukey´s multiple comparisons test, n=3). (**e**) Whole membrane images of GLI1 and GAPDH antibodies. Samples of interest blotted for GLI1, as illustrated on main Fig. 1d, are indicated by the black dotted frame.

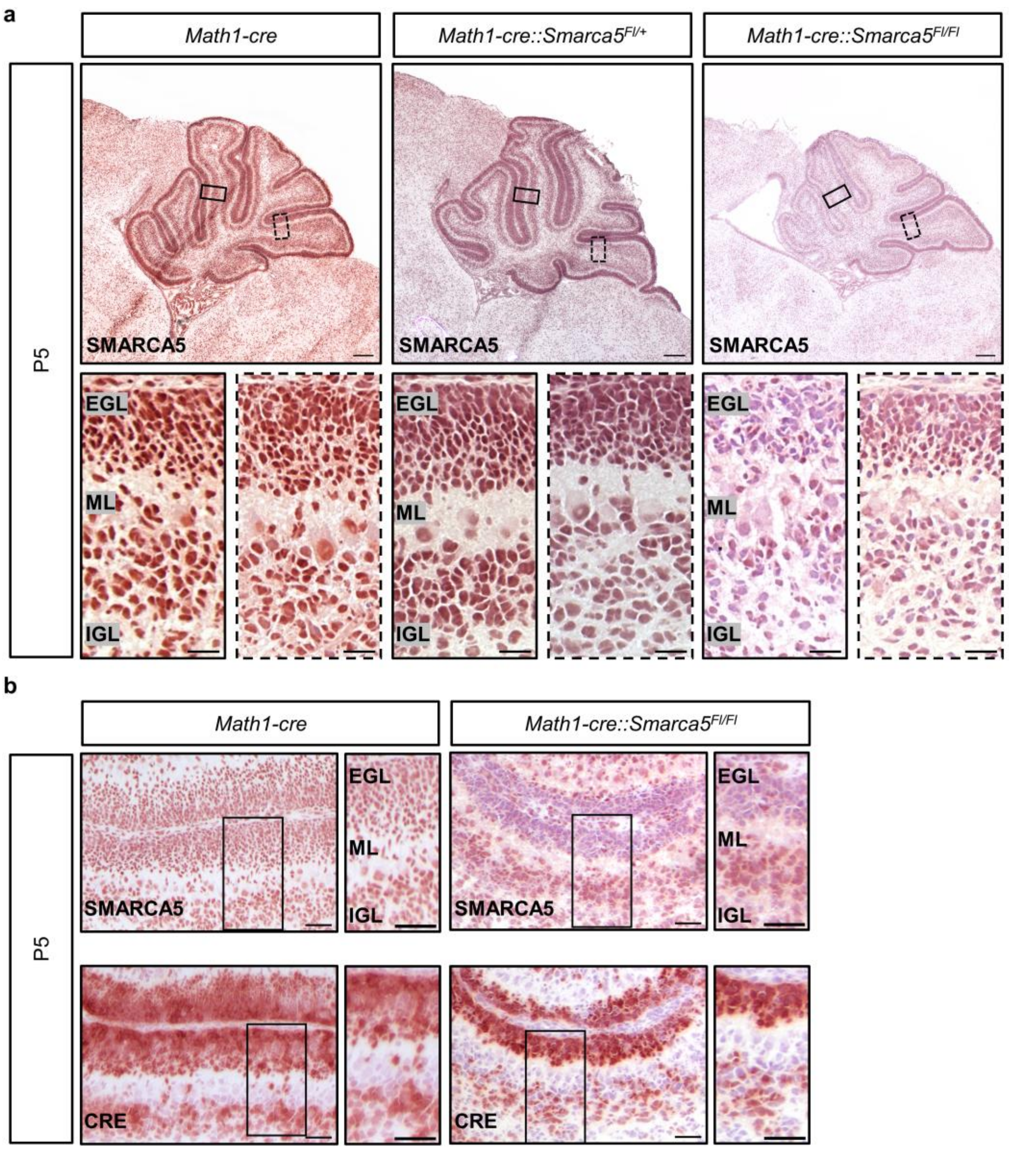

Figure S4. Histological analysis of *Math1-cre*, *Math1-cre::Smarca5*$^{Fl/+}$, and *Math1-cre::Smarca5*$^{Fl/Fl}$ mice at P5. (**a**) Sagittal sections from cerebella of *Math1-cre* (left panel), *Math1-cre::Smarca5*$^{Fl/+}$ (middle panel) and *Math1-cre::Smarca5*$^{Fl/Fl}$ (right panel) mice stained with SMARCA5 antibody at P5. Magnified boxes indicate anterior (solid black line, left side) and posterior (dotted black line right side) parts of the cerebellum. (**b**) Representative immunohistochemistry for SMARCA5 (upper panel) and CRE (lower panel) in the cerebellum of mice with indicated genotypes at P5. 4x magnification, scale bar, 500µm; 20x magnification, scale bar, 50µm. EGL, external granule layer; ML, molecular layer; IGL, internal granular layer.

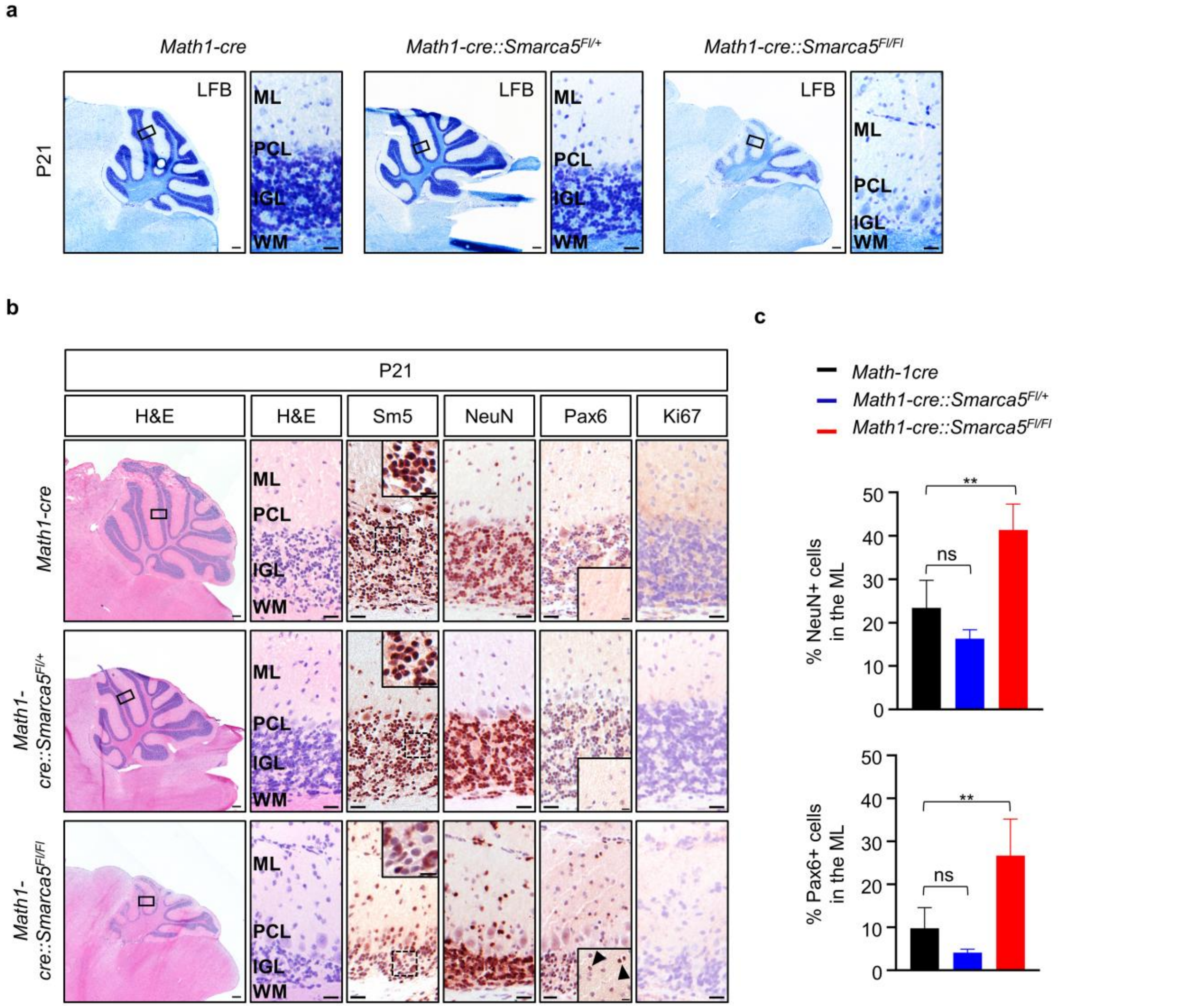

Figure S5. Histological analysis of Math1-cre, *Math1-cre::Smarca5*$^{Fl/+}$, and *Math1-cre::Smarca5*$^{Fl/Fl}$ mice at P21. (**a**) Representative cerebellar sections of *Math1-cre* (left panel), *Math1-cre::Smarca5*$^{Fl/+}$ (middle panel) and *Math1-cre::Smarca5*$^{Fl/Fl}$ (right panel) mice stained with Luxol Fast Blue (LFB). (**b**) Overview pictures of cerebella from mice shown in (a) stained with H&E, as well as for SMARCA5 (Sm5), NeuN, Pax6 and Ki67 markers. Arrowheads in Pax6 magnified panel indicate Pax6 positive cells. (**c**) Quantification of NeuN- (upper panel) and Pax6-positive cells (lower panel) in the molecular layer of cerebella from mice shown in (b) (n=3, Fisher´s exact test). 4x magnification, scale bar, 500µm; 20x magnification, scale bar, 50µm; 40x magnification, scale bar, 20µm. ML, molecular layer; PCL, Purkinje cell layer; IGL, internal granular layer; WM, white matter. All graphs display mean ± SD. ** p ≤ 0.01.

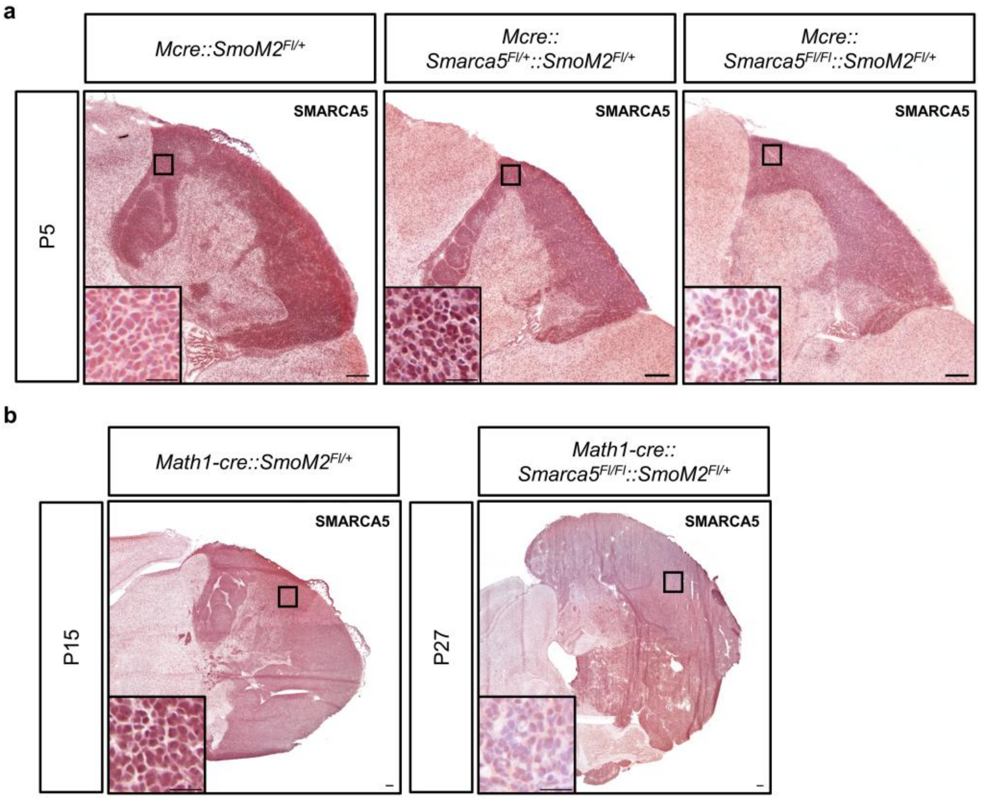

Figure S6. Histological analysis of *Math1-cre::SmoM2$^{Fl/+}$*, *Math1-cre::Smarca5$^{Fl/+}$::SmoM2$^{Fl/+}$*, and *Math1-cre::Smarca5$^{Fl/Fl}$::SmoM2$^{Fl/+}$* mice. Immunohistochemistry for SMARCA5 in whole cerebellar tumors from mice with indicated genotypes shown (**a**) in Fig. 2e and (**b**) in Fig. 2h. 4x magnification, scale bar, 500μm; 20x magnification, scale bar, 50μm.
